# Modification of the Bivalve Speculum

**Published:** 1852-06

**Authors:** Joseph Smith

**Affiliations:** Danville, Ky.


### *Modification of the Bivalve Speculum.* By JOSEPH SMITH, M.D., of Danville, Ky.

GENTLEMEN,—Having found considerable difficulty in using the bivalve speculum, on account of the protrusion of the soft parts between its blades when expanded, except by a modification of the instrument, as suggested by Dr. Bennet's work on the uterus, and not being able to get one so modified, a remedy suggested itself to me, which I have tried with complete success. It is to have a thin gum-elastic tube, or open sac, to enclose the speculum. It should be made very thin, and fit the instrument closely, when unexpanded. To do this, it must of course be larger at one end than the other. Not being near any gum-elastic establishment, I used a new tobacco pouch of this material. By cutting and sewing, one was made, which, though not so neat, has answered well. It does not increase the difficulty of introducing the instrument, and permits a free expansion of its blades.

I have not seen a suggestion to remedy this difficulty. If none has been made, I think you will confer a favor on many persons by publishing the above.

